# Diagnostic utility of thyroid scan and ultrasound in managing thyroglossal cysts: a systematic literature review

**DOI:** 10.25122/jml-2025-0006

**Published:** 2025-06

**Authors:** Wafaa Taishan, Mohammad Alessa, Majd Alsaleh, Turki Althunayyan, Rakan Almesned, Saleh Alessy, Hashem Alsaab, Jabir Alharbi, Sherif Abdelmonim, Ameen Alherabi, Haddad Alkaf, Mohammed Alqaddi, Ahmed Bahaj

**Affiliations:** 1 Department of Otolaryngology, King Faisal Hospital, Mecca, Saudi Arabia; 2 Head and Neck & Skull Base Health Center, King Abdullah Medical City, Makkah, Saudi Arabia; 3 Department of Otorhinolaryngology, Aljabr Eye and ENT Hospital, Alhassa, Saudi Arabia; 4 College of Medicine, Qassim University, Qassim, Saudi Arabia; 5 Department of Diagnostic Radiology, King Abdulaziz Medical City, Ministry of National Guard Health Affairs, Riyadh, Saudi Arabia; 6 Public Health Department, College of Health Sciences, Saudi Electronic University, Riyadh, Saudi Arabia; 7 Division of Research & Innovation, King Faisal Specialist Hospital & Research Center, Riyadh, Saudi Arabia; 8 Department of Pharmaceutics and Pharmaceutical Technology, Taif University, Taif, Saudi Arabia; 9 Otolaryngology, Head and Neck Surgery Department, Faculty of Medicine, Ain Shams University, Cairo, Egypt; 10 Otolaryngology-Head & Neck Surgery, Umm Al-Qura University, Makkah, Saudi Arabia

**Keywords:** thyroglossal cyst, thyroid scan, ultrasound, diagnostic utility

## Abstract

Thyroglossal duct cysts (TGDCs) are the most common congenital neck masses, frequently diagnosed in both pediatric and adult populations. Accurate preoperative diagnosis and imaging are essential for effective management. Ultrasound (US) and thyroid scintigraphy are the primary imaging modalities used in clinical practice. This systematic review evaluates the diagnostic utility of US and thyroid scintigraphy in the management of TGDCs, comparing their diagnostic performance and assessing whether a combined imaging approach improves patient care. The review was conducted in accordance with the Preferred Reporting Items for Systematic Reviews and Meta-Analyses (PRISMA) guidelines. Relevant studies assessing the diagnostic roles of US and thyroid scintigraphy in TGDCs were identified through comprehensive searches of PubMed, Web of Science, Scopus, and ScienceDirect, with the final search conducted on May 5, 2024. A total of 18 studies involving 823 patients met the inclusion criteria. The results consistently showed that ultrasound is the preferred imaging modality, offering noninvasive, radiation-free, and highly accurate diagnostic capabilities. Ultrasound confirmed TGDC diagnosis in 66.1% of cases across studies, with detailed anatomical imaging supporting preoperative planning. Thyroid scintigraphy, while useful in cases of suspected ectopic thyroid tissue, was less frequently employed and generally unnecessary when a normal thyroid was identified on ultrasound. The Sistrunk procedure remains the preferred surgical intervention, with preoperative US proving essential in planning. Ultrasound is the most effective and noninvasive imaging tool for diagnosing TGDCs and planning preoperative interventions. Thyroid scintigraphy should be reserved for selected cases in which ectopic thyroid tissue is suspected. The findings support the use of ultrasound as the primary imaging modality, with thyroid scanning playing a secondary and more selective role in the management of TGDC.

## INTRODUCTION

The two main categories of anomalies in the cystic neck are congenital and acquired lesions [1]. Among these, thyroglossal duct cysts (TGDCs) are the most common congenital neck masses, accounting for approximately 7% of the global population, with equal prevalence in men and women [2-4]. TGDCs result from the remains of the embryological thyroid descent pathway [4,5]. Although TGDCs are typically benign and often present only cosmetic concerns, they can present a variety of clinical manifestations, including infection, inflammation, and, in rare cases (approximately 1%), malignant transformation [6]. Accurate diagnosis and appropriate care are crucial to prevent complications and enhance patient outcomes. A suitable history, physical examination, and pertinent radiological studies are required to diagnose thyroglossal duct cyst [1]. While thyroid ultrasound and scanning are commonly employed modalities, their specific roles and diagnostic utility in managing TGDCs remain a subject of debate. Existing literature offers inconsistent findings regarding the optimal imaging strategy for these cysts. Some studies have suggested that thyroid ultrasound is sufficient to characterize TGDCs and guide surgical management [7]. On the other hand, many support routine thyroid scans to rule out the presence of ectopic thyroid tissue inside the cyst, which may have an impact on the surgical plan [8]. The lack of consensus underscores the need for further research to clarify the diagnostic value of these imaging techniques in the management of TGDCs.

To address this knowledge gap, we systematically reviewed the available literature to identify all studies published to evaluate the diagnostic utility of thyroid ultrasound and scintigraphy in patients with TGDCs. By comparing the diagnostic performance of these modalities, we sought to determine whether one imaging technique is superior to the other or whether a combined approach is necessary for optimal patient care.

## MATERIAL AND METHODS

### Literature search strategy

This study was registered in the International Prospective Register of Systematic Reviews (PROSPERO; protocol ID: CRD42024543230) and conducted according to the Preferred Reporting Items for Systematic Reviews and Meta-Analyses (PRISMA) guidelines and Cochrane Review methodology [9,10]. A comprehensive literature search was conducted across four electronic databases—PubMed, Web of Science, Scopus, and ScienceDirect—with no restrictions on publication year to identify all relevant English-language studies. The final search was conducted on May 5, 2024. We searched for studies that utilized thyroid scans or ultrasound for diagnosing thyroglossal cysts, reporting on diagnostic accuracy and clinical outcomes. In addition to the database searches, we employed Google Scholar’s advanced search function, restricting the query to terms appearing in the title and abstract to identify original research articles and other relevant studies that may not have been indexed in the primary databases. We also performed a manual search of the reference lists of all included studies to identify any additional eligible articles. The MeSH terms and keywords used in the search strategy are listed in Table 1.

**Table 1:** Search terms (MeSH and keywords) used across databases

Database	
PubMed	("thyroid scan" OR "thyroid scintigraphy" OR "tc-99m thyroid scan" OR tc-99m scan OR ultrasound OR ultrasonography) AND ("Thyroglossal cyst" OR TDC OR TGDC OR TGDCs)
Web of Science	("thyroid scan" OR "thyroid scintigraphy" OR "tc-99m thyroid scan" OR tc-99m scan OR ultrasound OR ultrasonography) AND ("Thyroglossal duct cyst" OR TDC OR TGDC OR TGDCs)
Scopus	
ScienceDirect	("thyroid scan" OR "thyroid scintigraphy" OR "tc-99m thyroid scan" OR tc-99m scan OR ultrasound) AND ("Thyroglossal duct cyst" OR TDC OR TGDC OR TGDCs)

### Study selection

This study included only original articles that focused on utilizing thyroid scans or ultrasound for diagnosing thyroglossal cysts, reporting on diagnostic accuracy and clinical outcomes, or studies reporting outcomes of interest relevant to the clinical questions. Exclusion criteria included non-English language studies, studies that did not report outcomes of interest, studies that did not utilize ultrasound or thyroid scintigraphy in diagnosing TGDCs, and studies focusing on diagnostic modalities other than ultrasound or thyroid scan. Additionally, non-original articles (e.g., editorials, letters, commentaries, reviews, case reports, or series) were excluded from the analysis. Two authors, TA and WT, independently screened the titles and abstracts of the identified articles and reviewed the full text for potentially eligible studies. Articles were extracted from the databases and screened using Rayyan software [11]. In cases where the title and abstract did not provide sufficient information, the full text was reviewed. Any disagreements were resolved through discussion or consultation with a third author (MA).

### Data extraction

Data were independently extracted by two authors using a standardized data extraction form developed in Microsoft Excel. Extraction was performed by two authors from the text, tables, and figures of the included studies using a pre-designed, standardized extraction form. To ensure the reliability and accuracy of the extracted data, a second author independently reviewed the data extraction process and cross-checked all the extracted data points against the source materials to identify any discrepancies or missing information. This included crucial data such as study characteristics (author, year of publication, study design, country of origin, total patients, imaging modality, aims, results, and conclusions of each study), participant characteristics (age, sex, preoperative infection, and symptoms of the cysts), cyst characteristics (site of cyst, relation to hyoid bone, presentation, imaging modality, other investigations that were used in the diagnosis, and ultrasound features), and intervention characteristics (type of operation). Any discrepancies in data extraction were resolved by discussion or consultation with a third author.

### Assessment of bias

Two reviewers independently used the Methodological Index for Non-Randomized Studies (MINORS) to assess both retrospective and prospective non-randomized studies. MINORS is a validated 12-item tool specifically designed to evaluate the methodological quality of non-randomized surgical studies [12]. For cross-sectional studies, quality appraisal was conducted using the Appraisal Tool for Cross-Sectional Studies (AXIS), which is a descriptive assessment instrument used to critically evaluate cross-sectional survey research [13]. Each included study was evaluated using the appropriate tool (MINORS or AXIS).

## RESULTS

**Figure 1:**
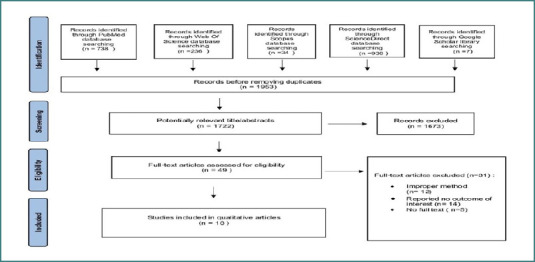
PRISMA flow chart for the systematic review

This systematic review identified a total of 1,953 published articles, comprising 738 articles from PubMed, 236 from Web of Science, 34 from Scopus, 938 from ScienceDirect, and seven articles from the Google Scholar library. After removing duplicates, 1722 articles were reviewed. We initially retrieved 49 full-text articles. However, after applying the predefined inclusion and exclusion criteria, 18 studies comprising a total of 823 patients were included in the final analysis (Figure 1). A total of 31 full-text articles were excluded for the following reasons: inappropriate study type (e.g., systematic reviews, review articles, letters to the editor, case reports; *n* = 12), no outcomes of interest reported (*n* = 14); and the full text was not available (*n* = 5).

The AXIS assessment revealed that the two cross-sectional studies had total scores of 15 and 17 out of a maximum of 22 points [13]. Overall, the included cross-sectional studies had a moderate risk of bias and moderate methodological quality, and the findings should be interpreted with caution, as shown in Supplementary Table 1.

Supplementary Table

MINORS assessment revealed that the included studies had total scores ranging from 10 to 12 out of a possible 16 points [12]. The most common methodological limitations were related to items 6, 7, and 8, where most studies received a score of 0. Overall, the methodological quality of the included articles was moderate, and the findings should be interpreted with confidence, as shown in Table 2.

**Table 2:** MINORS risk assessment tool

Item	[14]	[15]	[16]	[8]	[17]	[18]	[19]	[7]	[2]	[20]	[21]	[22]	[23]	[24]	[25]	[26]
1. A clearly stated aim	2	2	2	2	2	2	2	2	2	2	2	2	2	2	2	2
2. Inclusion of consecutive patients	2	2	2	2	2	2	2	2	2	2	2	2	2	2	2	2
3. Prospective collection of data	2	2	2	2	2	2	2	2	2	2	2	2	2	2	2	2
4. Endpoints appropriate to the aim of the study	2	2	2	2	2	2	2	2	2	2	2	2	2	2	2	2
5. Unbiased assessment of the study endpoint	2	2	2	2	2	2	2	2	2	2	2	2	2	2	2	2
6. Follow-up period appropriate to the aim of the study	0	0	0	0	0	0	0	2	0	0	2	0	0	0	2	0
7. Loss to follow up less than 5%	0	0	0	0	0	0	0	0	0	0	0	0	0	0	0	0
8. Prospective calculation of the study size	0	0	0	0	0	0	0	0	0	0	0	0	0	0	0	0
Total score	10	10	10	10	10	10	10	12	10	10	12	10	10	10	12	10

The MINORS tool scores for the included studies were based on individual item ratings of 0 (not reported), 1 (reported but inadequate), or 2 (reported and adequate). The ideal global score was 16 for non-comparative studies and 24 for comparative studies.

The studies reviewed in Table 3 provide a broad overview of the effectiveness of ultrasound and thyroid scintigraphy in diagnosing and managing thyroglossal duct cysts. Across nine studies, ultrasound consistently demonstrated its utility as a first-line imaging modality for detecting congenital cystic neck masses, particularly TGDCs. Most studies, such as those by Salahuddin e *t al*. [14] and Podzimek *et al*. [27], have concluded that ultrasound offers detailed imaging of TGDCs without the need for radiation, with findings that are well correlated with histopathological results. Key sonographic features, such as thin-walled, unilocular cysts and posterior acoustic enhancement, were recurrent across studies. In contrast, thyroid scintigraphy was employed selectively to confirm the presence or absence of ectopic thyroid tissue, as seen in Elmadani *et al*. [15] and Kessler *et al*. [8], although it was not deemed necessary if the thyroid was normal on ultrasound. Overall, ultrasound proved to be highly accurate and noninvasive, making it the preferred modality in preoperative assessments. It not only provided a comprehensive understanding of the structure of the cyst but also informed surgical planning, particularly for the Sistrunk procedure, which was the most common surgical approach across studies. Complex patterns seen in adult TGDC cases and variations in sonographic presentations further emphasize the importance of ultrasound in differentiating TGDCs from other neck masses [17]. These data support the consensus that while thyroid scans may be useful in specific cases, ultrasound alone is often sufficient for diagnosis and surgical management, consistent with findings from multiple studies.

**Table 3:** Diagnostic utility of ultrasound and thyroid scan in thyroglossal duct cysts

Name Author	Year	Country	Study Design	Total Patients	Imaging Modality	Result	Conclusion
Salahuddin [27]	2020	Khulna	Cross-sectional	50 (19 TGDC)	US	17/19 TGDC confirmed by cytology	USG is useful for diagnosing congenital cystic neck masses, correlating well with histopathology.
Podzimek [14]	2024	Germany	Retrospective observational	50	US	Detected TGDC with concerns for thyroid cancer in one case	HR ultrasound effectively detects TGDC and informs surgical planning.
Elmadani [15]	2010	Sudan	Retrospective cross-sectional	56	Thyroid scintigraphy, US	Both methods diagnosed 53 TGDCs, US more detailed	US is more detailed, with no radiation exposure, should be first-line imaging.
Wadsworth [16]	1994	Not mentioned	Retrospective	12	US	Sonograms varied from simple cysts to complex lesions	TGDC sonographic features vary; recognition aids in diagnosis.
Kessler [8]	2001	Israel	Retrospective	100	Thyroid scintigraphy, US	No ectopic thyroid tissue found	US confirms thyroid; scintigraphy reserved for select cases.
Kutuya [17]	2008	Japan	Retrospective	36	US	77.8% midline, 86.1% unilocular, 75% thin-walled	Inflammation affects sonographic features, typical for children’s TGDCs.
Ahuja [18]	1999	Hong Kong	Retrospective observational	40	US	88% posterior enhancement, 50% thin-walled	TGDCs in adults show complex sonographic patterns beyond simple cysts.
Tanphaichitr [19]	2012	USA, Chicago	Retrospective	122 (59 TGDC)	US	66.1% accuracy for TGDC, 30% for lymph nodes	US is useful in surgical planning but less accurate for pathological details.
Gupta [7]	2001	USA, Chicago	Retrospective	45	US	US identified TGDC in 37 cases, 2 dermoid cysts	US is accurate, non-invasive, no need for scintigraphy if normal thyroid found.
Ahuja [2]	2005	Hong Kong	Retrospective	23	US	Three patterns of TDCs were identified: anechoic (13%), pseudo-solid (56.5%), and heterogeneous (30.5%). Most were located midline (82.6%), with posterior enhancement (56.5%) and thin walls (82.6%).	TGDCs in children show complex sonographic patterns, ranging from anechoic to pseudo-solid forms.
de Jong [20]	1993	Netherlands	Retrospective	24	US, FNA, CT	Ultrasound accurately diagnosed TGDCs in 15 out of 17 confirmed cases. It can also detect asymptomatic and non-palpable TGDCs, indicating these conditions might be more common than previously believed.	Ultrasound is an effective diagnostic tool for detecting TGDCs and can also identify recurrences after surgery, as well as asymptomatic TGDCs.
Joseph [33]	2012	UK	Cross-sectional	194	US, TFT, TS, FNA, CT	95% of cases used US, and 32% also performed thyroid function tests. In 15% of cases, normal thyroid tissue was absent, and in 64% of these cases, this was the only functioning thyroid tissue.	There is a shift in clinical practice, with most relying on US for TGDC diagnosis. Ectopic thyroid tissue may be more common than previously thought.
Lim-Dunham [21]	1995	-	Prospective observational study	30	US	All patients had a normal thyroid on US.	Preoperative US showing a normal thyroid confirms the absence of ectopic thyroid tissue, eliminating the need for routine thyroid scintigraphy.
Bhatia [22]	2010	Hong Kong	Prospective observational study	5	US	TGDCs typically showed a unilocular, thin-walled cyst in the midline or slightly off-midline. Elastography showed a trend in stiffness; TGDCs and similar masses were generally soft.	Real-time qualitative elastography is a feasible technique for evaluating non-nodal neck masses but requires further research to overcome technical obstacles before routine clinical use.
Petrović [23]	2005	Serbia	Retrospective observational study	27	US	TGDCs had variable sonographic appearances: 48% pseudo-solid, 19% homogeneously hypoechoic, 9% heterogeneous, and 24% anechoic.	US is highly sensitive and accurate for differentiating congenital neck masses in children and is cost-effective for preoperative assessment.
Sherman [24]	1985	USA, Philadelphia	Prospective observational study	2	US, thyroid scan, FNA	US localized the mass and showed its relationship to the thyroid gland, trachea, and major neck vessels.	US is effective in localizing congenital neck masses and distinguishing their relationship to nearby structures.
Mettias [25]	2023	UK	Retrospective observational study	95	US, TFT, FNA, CT, MRI	85% underwent US, which showed a normal thyroid gland in all cases.	Preoperative US is the standard for evaluating TGDCs and ensures proper surgical planning while minimizing risk.
Kraus [26]	1985	-	Retrospective	6	US	TGDCs presented as cystic masses in the midline or slightly off-center.	US is valuable for assessing pediatric neck masses, with many having distinctive features allowing for accurate diagnosis.

Table 4 highlights the ultrasound features and clinical presentations of TGDCs in the reviewed studies. Ultrasound findings were consistently detailed, identifying TGDCs as well-defined, thin-walled, anechoic, or hypoechoic cysts, often showing posterior enhancement. In some cases, debris and septation were observed within the cyst, as reported by Salahuddin *et al*. [27]. The presence of inflammation, particularly in pediatric patients, has been noted in several studies, such as Kutuya *et al.*, where 19.4% of cases showed signs of infection [17].

**Table 4:** Preoperative ultrasound features and clinical characteristics of TGDCs

Study ID	*n*	Age (Mean ± SD)	Gender (*N*)	Site of cyst	Relation to hyoid bone	Presentation	Symptoms	Imaging modality & other investigations	Ultrasound feature	Pre-operative infection	Type of operation
Salahuddin, 2018 [27]	50 (19 TDC)	14.2 ± 1.33 years	-	Midline (19 cases)	-	Typical	Mass	US	Thin-walled, unilocular, anechoic or hypoechoic with debris and septation	No	-
Podzimek, 2024 [14]	50	3 to 83 years	16 M 34 F	Right/Left, 4 both sides of hyoid	8 superior, 38 typical	Typical	Mass	US	Well-defined margins, anechoic	No	45/50 had Sistrunk
Elmadani, 2010 [15]	56	Median 12.5 years	26 M 30 F	TGDC separate from thyroid in 53 cases	-	Typical	Mass	US confirmed TGDC separate from thyroid	No solid component, 2.1 cm average diameter	No	-
Wadsworth, 1994 [16]	12	Mean 6 years	4 M 8 F	Midline	All cases	Typical	Mass	US	Anechoic or hypoechoic, varying thickness	No	-
A Kessler, 2001 [8]	100	Mean 5 years	-	-	-	Typical	Mass	US	No ectopic thyroid	No	100/100 had Sistrunk
Kutuya, 2008 [17]	36	7.1 ± 4.6 years	19 M 17 F	Supra/intra/infra hyoid	6 supra, 16 at hyoid	Typical	Mass	US	25% anechoic, 41.6% heterogeneous, mean size 1.6 cm	19.4% with inflammation	-
Ahuja, 1999 [18]	40	-	-	Supra/infra hyoid	83% infrahyoid	Typical	Mass	US	Well-defined, anechoic or pseudo solid	No	-
Tanphaichitr, 2012 [19]	122 (59 TDC)	5.41 years	63 M 59 F	Midline	-	Typical	Mass	US	Anechoic or hypoechoic, thin-walled	Yes, 1 case	-
Gupta, 2001 [7]	45	-	-	Midline	TGDC	Typical	Mass	US	Anechoic, well-defined margins	No	-
Ahuja, 2005 [2]	23	2 to 15 years	12 M 11 F	Supra/infra hyoid	83% infrahyoid	Typical	Mass	US	Well-defined, thin-walled, anechoic to pseudo solid	No	-
de Jong, 1993 [20]	24	-	-	Midline	Close to hyoid in 8 cases	Typical	Mass	US	Anechoic, well-defined capsule	Yes, 17 cases	-
Joseph, 2012 [33]	194	-	-	-	-	Typical	Mass	US	Posterior enhancement	Yes	-
Lim-Dunham , 1995 [21]	30	Mean 4.2 years	22 M 8 F	Supra/infra hyoid	6 cases at base of tongue	Typical	Mass	US	Anechoic to homogeneously echogenic	No	-
Bhatia, 2010 [22]	49 (5 TDC)	42.8 years	20 M 29 F	Midline	TGDC	Typical	Mass	US	Thin-walled, anechoic	No	-
Petrović, 2005 [23]	53 (10 TDC)	3 months to 15 years	-	Midline	TGDC	Typical	Mass	US	48% pseudo solid	Yes, 1 case	-
Sherman, 1985 [24]	34 (2 TDC)	5.5 and 16 years	1 M 1 F	Right of thyroid	Superior to thyroid lobe	Atypical	Complex mass	US	Complex, elongated cystic mass	Yes, 2 cases	-
Mettias, 2023 [25]	95	Mean 24.7 years	45 M 45 F	Midline	61% supra hyoid	Typical	Mass	US	Posterior enhancement, thin-walled	No	81/95 had Sistrunk
Kraus, 1985 [26]	49 (6 TDC)	3 weeks to 18 years	-	Midline	TGDC	Typical	Mass	US	Anechoic, well-defined	Yes, all 6 cases	-

Preoperative infection was documented in varying degrees, with Mettias *et al*. [25] reporting infections in all six cases, while the majority of studies found no evidence of infection before surgery. Additionally, the relationship between TGDC and the hyoid bone was frequently observed, with a predominance of cases located at or near the hyoid, although cases above or below the hyoid were also noted, particularly in studies by Kutuya *et al*. [17] and Tanphaichitr *et al*. [19].

The Sistrunk procedure was the most common surgical treatment performed in most of the cases reviewed. These studies further confirmed that the high resolution and detailed imaging of the ultrasound provide critical insights into the cyst's structure and relation to surrounding tissues, facilitating better surgical outcomes. In atypical cases, such as those reported by Sherman *et al*. [24], complex, elongated cystic masses require careful diagnostic evaluation. Overall, ultrasound consistently proved effective in both the typical and complex presentations of TGDCs, demonstrating its importance as a diagnostic and preoperative tool.

## DISCUSSION

Thyroglossal duct cysts are the most common congenital neck masses, frequently diagnosed in both children and adults [28-30]. The primary aim of this systematic review was to evaluate the diagnostic utility of ultrasonography and thyroid scans in managing TGDCs preoperatively. Through the synthesis of findings from multiple studies, it is evident that ultrasound remains the preferred first-line imaging modality for the diagnosis and preoperative management of TGDC, whereas thyroid scans are reserved for more selective cases.

Ultrasonography has been established as a highly effective diagnostic modality for TGDCs. As demonstrated in several studies, it offers distinct advantages, including its non-invasive nature, lack of radiation exposure, and high resolution, which enable detailed imaging of cystic neck masses. Salahuddin *et al*. [27] demonstrated that ultrasound not only confirmed the diagnosis of TGDC in 17 of 19 cases but also closely correlated with histopathological findings, further supporting its accuracy. The noninvasive nature of ultrasound makes it a valuable tool in both pediatric and adult populations, where avoiding radiation is especially important [31-33].

The sonographic features of TGDCs have been well-documented in the reviewed studies. Typically, TGDCs present as thin-walled, unilocular cysts with anechoic or hypoechoic characteristics and posterior acoustic enhancement, as observed in multiple studies [17,19]. These features are consistent with congenital cystic neck masses, and their recognition is crucial for accurate diagnosis and differentiation from other neck masses, such as dermoid cysts, lymph nodes, and malignancies [34,35]. The ability of ultrasound to characterize the internal architecture of the cyst, such as the presence of debris, septations, or heterogeneous components, further enhances its diagnostic utility [18]. This was particularly important in the study by Kessler *et al*., where ultrasound identified no ectopic thyroid tissue in 100 patients, negating the need for thyroid scintigraphy in most cases [8].

Although thyroid scintigraphy has historically played a role in evaluating TGDCs, its use has diminished over time due to advancements in and the reliability of ultrasound [36]. Thyroid scans are primarily reserved for cases where there is clinical suspicion of ectopic thyroid tissue or when ultrasound findings are inconclusive [15]. The selective use of thyroid scintigraphy was demonstrated in Kessler's study, where it was employed to rule out ectopic thyroid tissue in patients with TGDC [8]. However, the study concluded that if a normal thyroid gland is confirmed on ultrasound, scintigraphy is largely unnecessary, a sentiment echoed by the multiple studies included in this review.

The superiority of ultrasound over thyroid scintigraphy is further supported by the detailed imaging provided without the need for radioactive material, as emphasized by Podzimek *et al*. [14]. Thyroid scintigraphy, which is effective in specific cases, poses potential risks owing to radiation exposure and is less preferred, especially in pediatric populations [27]. Moreover, scintigraphy provides less anatomical detail than ultrasound, making it less suitable for preoperative planning, where precise localization of the cyst and its relationship to the hyoid bone or surrounding structures is crucial [19, 36].

In addition to its diagnostic capabilities, ultrasound plays a critical role in preoperative planning for TGDC management [37-39]. The most commonly employed surgical technique for TGDCs is the Sistrunk procedure, which involves excision of the cyst along with a part of the hyoid bone to reduce the risk of recurrence [26]. The ability of ultrasound to provide detailed imaging of the cyst’s relationship to the hyoid bone is indispensable for ensuring the complete removal of the cyst and any associated tract during surgery [19]. This is particularly important in atypical or complex cases, as reported by Sherman *et al*., where the cyst may be elongated or located in unusual positions relative to the hyoid bone, necessitating more precise surgical planning [24].

Inflammation and infection of the TGDC, which can alter its sonographic appearance, are additional considerations during preoperative evaluation [35]. Kutuya *et al*. noted that 19.4% of cases presented with signs of inflammation, which could complicate surgery if not properly managed [17]. The presence of debris or septation within the cyst may indicate infection or previous episodes of inflammation, further emphasizing the need for accurate preoperative ultrasound assessment [25]. Early and accurate diagnosis of such cases allows appropriate medical management before surgical intervention, potentially reducing the risk of complications.

Despite the advantages of ultrasound, it has its limitations. One of the primary challenges of ultrasound is operator dependence; the accuracy and quality of the images obtained can vary based on the skill and experience of the ultrasonographer [7]. Additionally, while ultrasound is highly sensitive for detecting cystic structures, it may not always provide sufficient detail to evaluate potential malignancy within the TGDC, especially in cases with complex sonographic patterns [16]. This limitation underscores the need for histopathological confirmation following surgical excision, as noted by Salahuddin *et al*., who used cytology to confirm TGDC in 17 out of 19 cases [27]. Thyroid scintigraphy, although largely replaced by ultrasound, remains valuable in specific clinical scenarios. Its ability to detect ectopic thyroid tissue remains an advantage in cases where ultrasound findings are ambiguous or when there is a high clinical suspicion of ectopy [15]. However, its limited anatomical detail and reliance on radioactive material make it less suitable for routine preoperative assessment, particularly in pediatric populations, where minimizing radiation exposure is a priority [17].

### Clinical implications and future directions

The findings of this review have important implications for the clinical management of TGDCs. Ultrasound should be considered the first-line imaging modality for the diagnosis and preoperative assessment of TGDCs, given its accuracy, non-invasiveness, and ability to provide detailed anatomical information [19]. Thyroid scintigraphy, while useful in select cases, should be reserved for instances where ectopic thyroid tissue is suspected or when ultrasound findings are inconclusive [8].

Future research should focus on improving the diagnostic accuracy of ultrasound in detecting malignant transformation within TGDCs, as current imaging techniques may not always provide sufficient detail to assess the malignancy risk. Additionally, studies comparing the long-term outcomes of patients managed with ultrasound alone versus those treated with both ultrasound and scintigraphy could provide further insights into the optimal imaging approach for TGDCs.

## CONCLUSION

In conclusion, this systematic review highlights the diagnostic utility of ultrasound as the preferred imaging modality for TGDCs, supported by its detailed imaging capabilities and noninvasive nature. Thyroid scintigraphy remains valuable in specific cases but is generally unnecessary when a normal thyroid gland is confirmed on ultrasound. These findings support the continued use of ultrasonography as the primary tool for diagnosing and preoperatively managing TGDCs, thereby ensuring optimal patient care and surgical outcomes.
