# Supplementary material for: Diagnostic utility of thyroid scan and ultrasound in managing thyroglossal cysts: a systematic literature review
Source: J Med Life. 2025 Jun;18(6):517–25. doi: 10.25122/jml-2025-0006 (PMC12314846; doi:10.25122/jml-2025-0006)
Supplement: Supplementary file 1 [file JMedLife-18-517-s001.pdf]

Supplementary Table 1. Critical appraisal tool to assess the quality of cross-sectional studies (AXIS).

Item	Gazi Salahud-din, *et al.*	J Joseph, *et al.*
Aims/objectives clear	1	1
Appropriate study design	1	1
Justified sample size	0	1
Target/reference population defined	1	1
Representative sample frame	1	1
Representative selection process	1	1
Non-responders addressed	0	1
Appropriate risk factor/out-come variables	1	1
Correctly measured variables	1	1
Clear statistical significance/ precision	0	0
Sufficiently described methods	1	1
Adequately described data	1	1
Participant numbers reported	1	1
Participant characteristics described	1	1
Missing data reported	0	0
Internally consistent results	1	1
Analyses results presented	1	1
Justified discussions/conclu-sions	1	1
Study limitations discussed	0	0
Funding/COI affecting inter-pretation	0	0
Ethical approval/consent attained	0	0
Other strengths/weaknesses	1	1
Total score	15	17

The items are scored 0 (No), or 1 (Yes)

© 2025 by the authors. This article is an open access article distributed under the terms and conditions of the Creative Commons Attribution (CC BY 4.0) license.
